# Dynamics of microRNAs in bull spermatozoa

**DOI:** 10.1186/1477-7827-10-82

**Published:** 2012-09-14

**Authors:** Aruna Govindaraju, Alper Uzun, LaShonda Robertson, Mehmet O Atli, Abdullah Kaya, Einko Topper, Elizabeth A Crate, James Padbury, Andy Perkins, Erdogan Memili

**Affiliations:** 1 Departments of Animal and Dairy Sciences, Mississippi State University, Mississippi State, MS, 39762, USA; 2 Brown University, Providence, RI, 02912, USA; 3 University of Wisconsin Madison, Madison, WI, 53706, USA; 4 Department of Obstetrics and Gynecology, Faculty of Veterinary Medicine, Dicle University, Diyarbakir, Turkey; 5 Alta Genetics, Inc, Watertown, WI, 53094, USA; 6 New College of Florida, Sarasota, FL, USA; 7 Computer Sciences and Engineering, Mississippi State University, Mississippi State, MS, 39762, USA

**Keywords:** Uncompensatory fertility, Mammalian, Fertilization, Embryonic development

## Abstract

**Background:**

MicroRNAs are small non-coding RNAs that regulate gene expression and thus play important roles in mammalian development. However, the comprehensive lists of microRNAs, as well as, molecular mechanisms by which microRNAs regulate gene expression during gamete and embryo development are poorly defined. The objectives of this study were to determine microRNAs in bull sperm and predict their functions.

**Methods:**

To accomplish our objectives we isolated miRNAs from sperm of high and low fertility bulls, conducted microRNA microarray experiments and validated expression of a panel of microRNAs using real time RT-PCR. Bioinformatic approaches were carried out to identify regulated targets.

**Results:**

We demonstrated that an abundance of microRNAs were present in bovine spermatozoa, however, only seven were differentially expressed; hsa-aga-3155, -8197, -6727, -11796, -14189, -6125, -13659. The abundance of miRNAs in the spermatozoa and the differential expression in sperm from high *vs.* low fertility bulls suggests that the miRNAs possibly play important functions in the regulating mechanisms of bovine spermatozoa.

**Conclusion:**

Identification of specific microRNAs expressed in spermatozoa of bulls with different fertility phenotypes will help better understand mammalian gametogenesis and early development.

## Background

Fertilization is the most important factor controlling mammalian reproduction in which a developmentally competent spermatozoon fertilizes the egg and sets the stage for egg activation and embryo development. Even though producing an abundant amount of spermatozoa with normal motility and morphology, the fertility of some bulls is low which may be due to molecular defects in the sperm [1]. Molecular and cellular integrity of sperm is not only important for fertilization but also for embryo development and later fetal development. MicroRNAs are small non-coding RNAs that regulate gene expression of messenger RNA which are abundant in bull spermatozoa [2]. We asked: What are the microRNAs (miRNAs) that are present in the mature male gamete? What does their presence in the spermatozoa mean? What roles do these miRNAs play in fertilization, egg activation, early and later development? Answers to these fundamental questions remain unknown at the present.

Successful development is a highly choreographed process with precision in the timing and degree of gene expression at each step. The control of gene expression is exhibited at each point of the pathway from transcription to translation. MiRNAs are among the newly recognized factors associated with important post-transcriptional regulation of gene expression. As such, miRNAs have gained special interest and their roles during various developmental stages are the subject of many investigations. MiRNAs are tissue specific and they are involved in specific cell differentiation processes [3]. Discovery of candidate miRNAs will allow understanding of the regulatory pathway(s) of particular genes. Thereby, the spatial and temporal expression and even the function of individual genes can be defined.

MiRNAs are generated from double-stranded RNA precursors by Dicer endonucleases, and function with Argonaute-family proteins to target transcript-destruction or to silence translation.

It is known that developmentally regulated molecular events following fertilization include changes in the length of the cell cycles, remodeling of chromatin structure, DNA methylation, synthesis of zygotic/embryonic transcripts and proteins, and degradation of maternal transcripts [4,5]. Various studies have shown that miRNAs regulate gene expression and also play a major role in embryo development. These RNAs have been identified in a wide range of organisms, including bovine, and plants and are conserved through species such as: *C. elegans* through *D. melanogaster* to *Homo sapiens*[6]. The degree to which spermatozoa miRNAs regulate fertilization and early embryonic development in bovine has yet to be discovered.

Spermatogenesis is a multistep complex developmental process. It includes continuous cell proliferation and differentiation of germ cells into final form of functional spermatozoa. This differentiation process is dependent on the sequential expression of genes. However, molecular mechanisms underlying this regulation remain largely unknown. Numerous miRNAs in germ cells are subjected to post-transcriptional and translational regulation [7]. It would be significant to identify the sperm-specific miRNA and their target genes. This will help to better understand the molecular mechanisms by which an orderly sequence of germ cell differentiation takes place by switching on and off specific genes. Knowledge on the miRNA repertoire of sperm will also expand the scope of the search for functionality of sperm-originated small RNAs in oocyte activation, fertilization and subsequent embryonic development. It is known that differences exist among bulls in their ability to fertilize and activate the egg, and for the sperm to further support embryonic development [8]. The primary focus of this study is to identify miRNAs expressed in bovine spermatozoa bulls with differing fertility potential. We used a transcripome-wide strategy to compare the miRNA pools from low and high fertility bull spermatozoa and found seven differentially expressed miRNAs. Our results demonstrate that miRNAs are abundant in bovine spermatozoa. Furthermore, the levels of expression in miRNAs of high and low fertility bulls vary greatly and may be involved in controlling animal reproduction.

## Methods

We analyzed the expression of miRNAs from bovine spermatozoa in a high fertility bull and a low fertility bull. This was done by isolating the total RNA from sperm cells, enrichment of miRNAs, and performing microarray analysis. The results from microarray were validated by isolating sperm RNA and performing real time RT-PCR using sperm from additional bulls with varying fertility.

### Semen samples and data sources

Frozen semen samples and fertility phenotype from eight mature, progeny tested Holstein bulls with satisfactory semen quality were obtained from Alta Genetics (Watertown, WI). The two most extreme high and low fertility bulls were used for the sperm MicroRNA MicroArray Expression Array. The other six bulls were used for the validation of the results using reverse transcriptase real time PCR.

The fertility phenotypes have been characterized in the established progeny testing program (Alta Advantage®) which is the industry’s most reliable source of fertility information. This program consisted of 180 well managed partner dairy farms with an average of 850 milking cows each located in different geographical regions across the United States. The reproductive performance of the herds are consistently monitored through accurate registration of insemination records, plus, the breeding outcomes are diagnosed by veterinary palpation or ultrasound rather than relying on non-return rates 60-90 days after breeding. Additionally, the program provides the distinct advantage of verification of paternity of the offspring using DNA testing.

### Bull fertility prediction

The fertility of the sires is predicted and documented on a quarterly basis using updated results of the breeding records. For this study, the database used to predict bull fertility consisted of a total of 953,702 breeding records from 998 bulls. The breeding record for each bull was an average of 958 with a range of 300 to 12,312. In order to rank the bulls based on their breeding values for fertility, the environmental and herd management factors that influence the fertility performance were adjusted using threshold models which were similar to previously published models by Bartel and Zwald et al. [3,9]. Parameter estimation and fertility estimations were obtained using Probit.F90 software developed by Wanget al [10]. We considered the outcome of each breeding event and adjusted the environmental factors such as the effects of herd-year-month, parity, cow, days in milk, and sire’s proven status (young or proven). Then the fertility of each sire was expressed as the percent deviation of its conception rate from the average conception rates of all bulls in the database that have at least 300 breeding outcomes.

### Selection of high and low fertility bulls

We used standard deviation (SD) of the predicted conception rate as the threshold values to classify a bull as high and low fertility animals [11,12]. Furthermore, the bulls selected were from the group that had highest breeding records for high reliability. Briefly, to classify high or low fertility, we used two SD values above or below the average, indicating a four SD distance range between both groups, with a minimum of 700 breeding records for each individual bull. The average fertility of high and low fertility groups were 5.2 and -7.1 % of the average (Zero = 0), respectively. So this allowed a 12.3 % total fertility difference between high and low fertility groups. The average breeding for the high and low fertility groups were 1,419 ± 557 and 785 ± 122, respectively (Mean ± SD) (Table 1).

**Table 1:** Bull information for the samples used to validate the microarray studies using real time RT-PCR

**Bull no**	**Number of breedings**	**Fertility (% difference from average fertility)**	**Fertility status**
**1**	1723	4.4	HF
**2**	4,021	4.7	HF
**3**	779	4.4	HF
**4**	1,156	7.4	HF
**5**	722	−7.5	LF
**6**	747	−6.3	LF
**7**	704	−5.6	LF
**8**	967	−9.2	LF

### Isolation of spermatozoa

The sperm straws were thawed together in a water bath set to 36°C for 30 seconds. The sperm (20 x10^6^ cells) was poured (2 straws/tube) into the percoll gradient and spun at 1,509 x *g* for 15 minutes, in ambient air at 4°C in the centrifuge. Motile sperm cells were isolated using percoll gradient as described previously [13]. The supernatant, containing cryoprotectants and egg yolk, was carefully removed using a micropipette. The pellet was resuspended in 1 ml PBS and centrifuged again. The supernatant was again removed and the pellet was resuspended in 1 ml of PBS and centrifuged at 9,500 x *g* for 1 minute at 4°C. Following this final step, the supernatant was again removed, and the sperm pellet stored at -80^o^C.

### Sperm RNA extraction

RNA was isolated using *Trizol* (Invitrogen, Carlsbad, CA) according to Feugang et al. (2008) [12]. Briefly, 500 μl of *Trizol* were added into each sperm cell pellet all of which were then homogenized at high speed for 30 seconds using the Pro 200 homogenizer (Pro Scientific Inc., Oxford CT). Glycogen (3 μl of 20 mg/ml) was added to the tubes and another 500 μl of *Trizol* was then added followed by mixing by several pipettings. Then, the mixture was incubated for 15 minutes at 65°C. Finally five hundred microliters of chloroform were added and is mixed again by means of several pipettings followed by vortexing for 40 seconds.

The samples were incubated at room temperature for 10 minutes followed by centrifugation for 15 minutes at 4°C. The upper colorless phase was collected into a new sterile eppendorf tube into which five hundred microliters of 100% isopropanol were added and mixed by several pipettings.

Following incubation at -20°C for an hour, the samples were vortexed for 10 seconds and incubated at room temperature for 10 minutes. The RNA was precipitated by centrifugation for 10 minutes at 4°C. Following the removal of the supernatant, the pellet was air-dried for 5 minutes and then was rehydrated in 20 μl of deionized, diethylpyrocarbonate (DEPC) treated water. The concentration of the RNA was determined using 2 μl of the samples in Nanodrop ® ND 1000 (NanoDrop Technologies, Wilmington, DE). The remaining 18 μl of the samples were stored in -80^o^C freezer and then shipped to Asuragen Inc. on dry ice.

### Enrichment of spermatozoal microRNAs and microRNA microArray expression array experiments

Samples for miRNA profiling studies were processed by Asuragen Services [14], according to the company’s standard operating procedures. Total RNA was dephosphorylated with calf intestinal phosphatase and the pCp-Biotin labeling molecule was ligated to the 3’ ends of the RNA molecules. Labeled RNA was purified using BioSpin6 (Bio-Rad, Hercules CA). Hybridization, washing, staining, imaging, and signal extraction were performed according to Affymetrix-recommended procedures, except that the 20X GeneChip Eukaryotic Hybridization control cocktail was omitted from this hybridization procedure.

There were six samples processed for microarray experiments using the Ambion/Affymetrix DiscovArray. The signal processing implemented for the Ambion miRCHIP is a multi-step process involving probe specific signal detection calls, background estimate and correction, constant variance stabilization [15] and either array scaling or global normalization. For each probe, an estimated background value is subtracted that is derived from the median signal of a set of G-C-matched anti-genomic controls. Arrays within a specific analysis experiment were normalized together according to the variance stabilization method described by Huber et al. (2002) [15]. Detection calls were based on a Wilcoxon rank-sum test of the miRNA probe signal compared to the distribution of signals from GC-content matched anti-genomic probes.

### Statistical analysis

For statistical testing, a two-sample *t*-Test, with assumption of equal variance, was applied. One-way ANOVA was used for experimental designs with more than two experimental groupings or levels of the same factor. These tests define which probes are considered to be significantly differentially expressed, or "significant", based on a default p-value of 0.001 and log_2_ difference >1.

### Identification of miRNA target genes

First, we ranked the expression levels of miRNAs for high *vs.* low fertility bulls and low *vs.* high fertility bulls from high to low. We selected the top 10 from the list. Then we used the miRBase database to find the related sequence for each entry by using their precursor-accession IDs [16]. Sequences were entered into the BLAT program, UCSC [17], platform and possible target genes were identified. BLAT search is designed to find sequences of 95% and greater similarity, and of length 25 bases or more. We used the human genome as a template to identify the target genes. After identification of each gene we used the HUGO Gene Nomenclature Committee (HGNC) database for annotation purposes [18].In identification of seven differentially expressed miRNAs, again we followed the same steps and presented results in Table 2.

**Table 2:** Characterization of Seven Probe Sets used for validation of microarray results using real time-RT-PCR

**IDs**	**Chr**	**Sequences**	**P-values**	**Log Ratio**	**Fold change D*vs.*R**	**Target Gene**	**# of Physical Interactions**
hsa-aga-3155	22	AAGCUUAUGGAGCAGAGGAUU	0.00001	3.86	4.65	TOB2	14
hsa-aga-8197	7	UGAGUGAUAAUAGGGUCGUGAC	0.0004	1.22	5.6	CHN2	2
hsa-aga-6727	18	UUCUGUGGCAGAUUGGGAUGGA	0.00051	1.83	4.27	CLUL1	"-"
hsa-aga-11796	22	GAGGCAGAGAAGGGACAGGAAA	0.00073	1.72	7.35	BC035867	"-"
hsa-aga-14189	19	CAUAGCGAGACCCCGUCUG	0.00077	1.21	14.34	BTBD2*	3
hsa-aga-6125	9	UUGGAUAUUGCUUGGAGGCUCU	0.0009	1.25	10.32	No hits	"-"
hsa-aga-13659	9	GACUGGAGGAGGCAUGGAGGGU	0.0009	1.05	12.93	AQP7P1	"-"

### Determination of miRNA interactomes

We used the list of predicted target genes (TOB2, CHN2, CLUL1, BC035897, BTBD2, AQP7P1) for the seven differentially expressed miRNAs as an input to Genemania [19]. Genemania is a web based tool that searches many large, publicly available biological datasets. We searched for physical interactions which cover protein-protein interactions and genetic interactions. Genemania, which uses data from BioGRID Physical, predicted protein interaction data, based on orthology from I2D and pathway and molecular interaction data from Pathway Commons. We applied default parameters and a restriction on the results applicable only to *Homo sapiens.*

### Validation of the microarray data

#### Isolation of miRNAs from bull spermatozoa

Spermatozoa from eight bulls with varying fertility (Table 1) were isolated as described above. MicroRNAs were isolated from 50 million sperm cells using the *mir*VANA kit according to manufacturer's protocol (Ambion, Austin, TX). Briefly, sperm pellets were resuspended in the lysis/binding buffer and passed through a 25 G needle followed by addition of Acid:Phenol:Chloroform and miRNA homogenate additive. The sample was again passed through a 25 G needle and vortexed for 10 min. Following 10 min incubation on ice, the homogenized sample was centrifuged at 10,000 x *g* for 5 min at room temperature. Upper phase of the solution was carefully transferred into a new sterile tube and was mixed with 1/3 ratio (v/v) of 100% ethanol. The mixture was loaded to a filter cartridge and centrifuged for 15 sec at 10,000 x g at room temperature. The filtrate was collected in a fresh tube and mixed with 2/3 ratio (v/v) of 100% ethanol, loaded into a new filter cartridge and centrifuged for 15 sec at 10,000 x g. Subsequently, the filter cartridge was washed once with miRNA wash solution 1 and twice with miRNA washes solution 2. Following each washing step, the filter cartridge was centrifuged for 10 sec at 10,000 x *g*, and for one min to remove residual fluid from the filter at the last wash. Finally, the filter was transferred to a new collection tube and 50 μl of elution buffer pre-heated to 95^o^C was applied to the center of the filter and centrifuged for 30 sec at max speed to recover miRNA. Concentrations of miRNA were measured using the ND-1000 spectrophotometer (NanoDrop Technologies, Palo Alto, CA).

### Reverse transcriptase real time PCR

Prior to reverse transcriptase (RT) reaction, isolated miRNAs were digested with DNAse I (1U, Promega, WI) to remove possible genomic DNA contamination. Following the digestion, 30 pg of miRNA were polyA-tailed using poly (A) polymerase and then reverse-transcribed using miScript Reverse Transcription Kit from Qiagen (Valencia, CA) according to the manufacturer’s protocol. Briefly, the reaction was set up as following: 10 μl of 2X QuantiTect SYBR Green PCR Master Mix, 2μl of10X miScript Universal Primer, 2μl of 10X miScript Primer Assay (Specific Primer) 1.5 pg RT product (1μl cDNA) and ddH_2_O up to 20 μl of final volume. Thermal cyclic conditions were: initial activation step at 95°C for 15 min followed by 40 cycles of denaturation, annealing and amplification (94°C 15 sec, 55°C 30 sec, 70°C 30 sec) on DNA Engine Opticon 2 Real-Time PCR Detection System (Bio-RAD, Foster City, CA). Melting curve analysis was performed as following: 95°C for 1 min, then fluorescence measurement was done at every 0.8-degree increments between 60°C and 95°C. In each run, a negative control with no cDNA template was included. All samples were run in duplicate for each cDNA isolation. Five different miRNAs (RNU1A, RNU6B, RNU5A, SNORD25, and SNORA73A) in the miScript PCR control set (Qiagen) were employed for normalization of miRNA expressions. The real time RT-PCR experiments were repeated three times using three independent miRNAs.

### Statistical analyses of the real time RT-PCR

The amounts of each isolated miRNAs for high fertility and low fertility bulls were compared by *t*-test. Data were presented as mean ± SEM. Differences were considered significant when P values were lower than 0.05. Regarding statistical analyses of the qPCR data, cycle threshold [20] values of high fertility bulls and low fertility bulls were normalized by using five reference miRNAs (RNU1A, RNU6B, RNU5A, SNORD25, SNORA73A). Normalized values for high fertility and low fertility groups were compared by using the algorithm (Relative Expression Software Tool 2009) upon which the mathematical model is based. This basis depends on the PCR efficiency of each gene investigated and the mean deviation in Ct between groups [21]. The expression ratios were considered statistically significant at p < 0.05.

## Results

### MicroRNAs of bull spermatozoa

**Figure 1:**
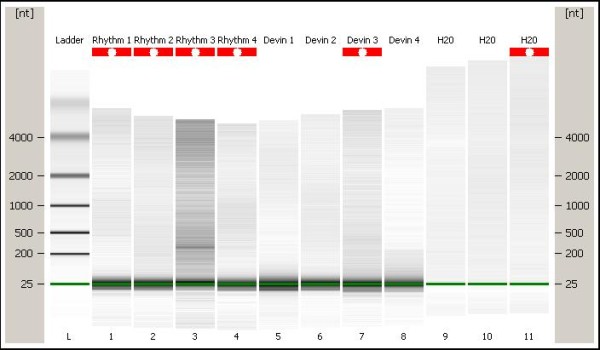
**Small molecular weight RNA transcripts as detected using Bioanalyzer.** According to the gel image there is no contamination in the RNA and the miRNA will be at or below the green line on the gel, given that miRNAs range from 17-25 nt in length. Rhythm 1, Rhythm 2, Rhythm 3, and Rhythm 4 represent biological repeats for low fertility bull of -3.3 ± 3.3 fertility %. Devin 1, Devin 2, Devin 3, and Devin 4 represent biological repeats for high fertility bull of 4 ± 1.8 fertility %.

To confirm the presence of abundant miRNAs in bull spermatozoa, the Bioanalyzer was used to determine the purity of RNA and ensure that miRNAs were indeed present in the mature sperm. For each bull there were three sample repeats and the bioanalyzer showed that small RNAs were present in each bull and their repeats (Figure 1). Small RNAs which include microRNAs were visible at 25 nt on the gel showing an abundance of small RNAs based on the size of the band. Absence of ribosomal RNA bands suggests that the sperm RNA samples were somatic cell free.

### Dynamics of microRNAs in bull spermatozoa

**Figure 2:**
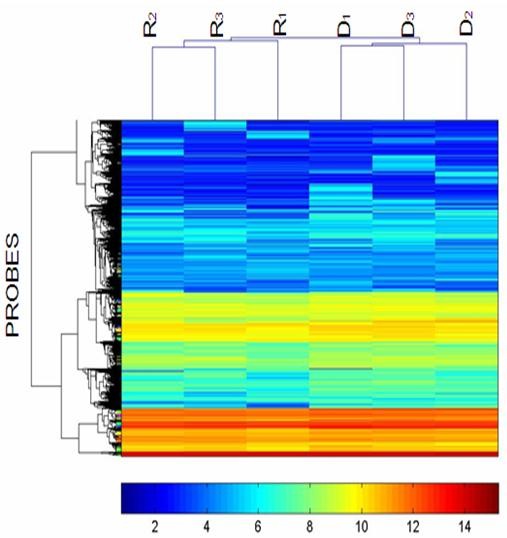
**Heat map and the result of clustering to all MicroRNA probes detected.** There are two specific groups. Column 1-3 represents bull R. Columns 4-6 represents bull D. There were differences between the two groups and some differences within the same groups.

The miRNA samples were analyzed using a miRNA microarray (Ambion/Affymetrix DiscovArray) containing several types of probe sets derived from organisms such as plants (i.e. rock cress and soybean) and animals (i.e., zebra fish, chicken, mouse, and human). The bulls were categorized as either a high (D) or a low (R) fertility bull. Each sample is represented by a vector containing all detected probes (14215 probes). The samples showed that there were general differences between the high and low fertility bulls (Figure 2). It also, showed that there were differences among the same sample. Approximately 2510 probe sets were detected significantly above background (See Additional file 1: Table S1).

### Statistical analysis of differentially expressed microRNAs

A heat map of detected probes is shown in Figure 2. The single horizontal red line corresponds to a non-adjusted p-value of 0.05. Genes above this line were considered statistically significant at a p-value of 0.05. The expression of high fertility bull (D) is compared to that of low fertility bull (R). The highly differentially expressed genes are indicated by the lines colored red. There are seven of these genes that are called significant based on an unadjusted p-value of 0.001 and a 2-fold change (Figure 2).

Each sample is represented by a vector containing only significant probes. This reduction eliminates the influence of non-significant probes to the cluster analysis. Each probe is represented in each line, with the color code confirming their differential expression levels between low and high (D) fertility bulls, despite the slight variation between replicates in each bull. Characterizations of the seven probe sets are summarized in Table 2. All the probes are from humans and their annotations are not yet completed.

### Identification of miRNA target genes

In our top 10 miRNAs some probes are not human based so that we could not identify the target genes for non-human miRNAs (Tables 3 and 4). We identified several genes in the top 10 list, namely, DALRD3, C21orf34, AK091713, DNM2, and IFT80. In summary, gene ontology (GO) terms are reported for DALRD3, showing that it is related to ATP binding and nucleotide binding [22]. DNM2 has a role in biological processes such as DNA-dependent regulation of transcription, signal transduction, and/or receptor internalization. IFT80 is a protein component of intraflagellar transport (IFT) complex B, and it is required for the formation, maintenance and functionality of cilia. We also compared our findings that are presented in Tables 3 and 4 with a recent study from Krawetz et al. [23]. In Tables 3 and 4, hsa-miR-191 was also found in the Krawetz et al. study. It was associated with genomics features; a TSS/Promoter and histones. It had been previously identified in sperm, testis, ovary, zygote and it was also reported that hsa-miR-191 was not regulated epigenetically. Another microRNA, i.e., hsa-miR-100, was also found to be associated with genomic features; TSS/Promoter. It was previously identified in testis, ovary, and zygote. It was found that hsa-miR-100 is epigenetically regulated.

**Table 3:** **Top 10 miRNAs expressed in high fertility bull (D) *vs. *low fertility bull (R)**

**Probe_ID**	**MicroRNA Accession**	**Precursor Accession**	**D: Mean**	**R: Mean**	**D *vs. *R: test**	**Target gene**	**Function of target gene products**
hsa-miR-191_st2	MIMAT0000440	MI0000465	13.95686623	13.02345008	0.01791063	DALRD3	DALR anticodon binding domain containing 3
hsa-miR-125b_st1	MIMAT0000423	MI0000470	13.91045299	13.02384618	0.01402603	C21orf34	
ame-miR-125_st1	MIMAT0001474	MI0001578	13.90178917	12.94703119	0.01176129	NI*	
hsa-cand375_st1	MIMAT0000098	MI0000102	13.81001246	13.37153817	0.13644647	AK091713	
hsa-miR-638_st2	MIMAT0003308	MI0003653	13.78857283	13.96774448	0.0187295	DNM2	dynamin 2
hsa-miR-100_st2	MIMAT0000098	MI0000102	13.59036572	13.0028788	0.07920135	AK091713	
dps-miR-289_st1	MIMAT0001253	MI0001348	13.21245856	13.84341616	0.0205619	NI*	
hsa-miR-15b_st1	MIMAT0000417	MI0000438	13.19413812	11.97871936	0.07635284	IFT80	intraflagellar transport 80 homolog
dme-miR-100_st1	MIMAT0000357	MI0000378	12.89301651	12.12274822	0.05917641	NI*	
dre-miR-15b_st1	MIMAT0001773	MI0001893	12.66899194	11.24401476	0.04350151	NI*	

NI* Not Identified.

**Table 4:** **Top 10 miRNAs expressed in low fertility bull (R) *vs. *high fertility bull (D)**

**Probe_ID**	**MicroRNA Accession**	**Precursor Accession**	**D: Mean**	**R: Mean**	**D *vs. *R: ttest**	**Target Gene**	**Function of target gene products**
hsa-miR-638_st2	MIMAT0003308	MI0003653	13.78857283	13.96774448	0.0187295	DNM2	dynamin 2
dps-miR-289_st1	MIMAT0001253	MI0001348	13.21245856	13.84341616	0.0205619	NI*	
hsa-cand375_st1	MIMAT0000098	MI0000102	13.81001246	13.37153817	0.13644647	AK091713	
hsa-miR-125b_st1	MIMAT0000423	MI0000470	13.91045299	13.02384618	0.01402603	NI*	
hsa-miR-191_st2	MIMAT0000440	MI0000465	13.95686623	13.02345008	0.01791063	C21orf34	
hsa-miR-100_st2	MIMAT0000098	MI0000102	13.59036572	13.0028788	0.07920135	AK091713	
hcmv-miR-UL70-3p_st2	MIMAT0003343	MI0003688	12.62649632	12.99272922	0.00473263	NI*	
ame-miR-125_st1	MIMAT0001474	MI0001578	13.90178917	12.94703119	0.01176129	NI*	
dme-miR-100_st1	MIMAT0000357	MI0000378	12.89301651	12.12274822	0.05917641	NI*	
hsa-miR-15b_st1	MIMAT0000417	MI0000438	13.19413812	11.97871936	0.07635284	IFT80	intraflagellar transport 80 homolog

NI* Not Identified.

Among the seven differentially expressed miRNAs from the array we were able to verify six of them and these are TOB2, CHN2, CLUL1, BC035897, BTBD2, and AQP7P1. The first, TOB2 belongs to family of antiproliferative proteins, which are involved in the regulation of cell cycle progression [24]. Like the TOB protein, TOB2 inhibits cell cycle progression from the G0/G1 to S phases. CHN2 has GTPase-activating protein activity that plays a significant role in the proliferation and migration of smooth muscle cells. CLUL1 is related with cell death. BTBD2 protein binds Topoisomerase I. In addition, it is stated in the literature that polymorphisms of LIG4, BTBD2, HMGA2, and RTEL1 genes are involved in the double-strand break repair pathway (PMID 20368557).

### Determination of miRNA interactomes

**Figure 3:**
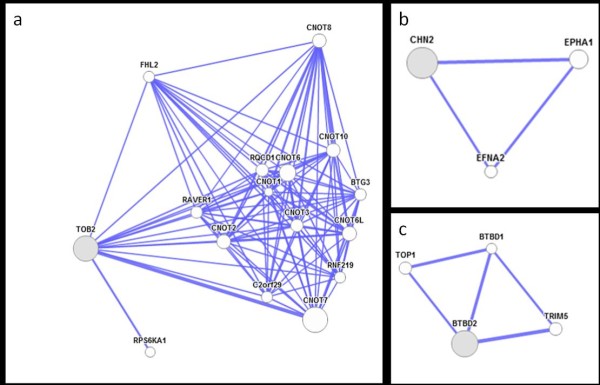
**Interactome of miRNA targets.** Figures present predicted physical (protein-protein) interactions of a) TOB2 b) CHN2 c) BTBD2.

Gene networks that are composed of miRNAs and their target mRNAs may represent important possible biological functions. In our analysis we found interactions for 3 genes from the list above, they are TOB1, CHN2 and BTBD2. In addition, TOB2 has 12 possible protein-protein interactions. Two of these interactions represent stronger associations than the rest and these two most significant interactions occur between TOB2-CNOT7 and TOB2-RPS6KA1 as shown in Figure 3A. TOB2 is a transducer of ERBB2 and belongs to the TOB/BTG1 family of antiproliferative proteins that are involved in the regulation of cell cycle progression. Another significant finding about CHN2 is shown in Figure 3B. This gene has interactions with EPHA1 and EFNA2. Both EPHA1 and EFNA2 have been implicated in mediating developmental events, particularly in the nervous system. BTBD2 has physical interactions between TOP1, BTBD1and TRIM5 as indicated in Figure 3C.

### Validation of the microarray data using real time RT-PCR

Total amounts of miRNAs isolated from 50 million sperm cells from the six bulls ranged between 148.6 ± 9.6 and173.3 ± 26.3 which shows there were not significant differences among the bulls. Optic density of 260/280 in the Nanodrop measurement was 1.7 ± 0.1 for the isolated miRNAs.

**Figure 4:**
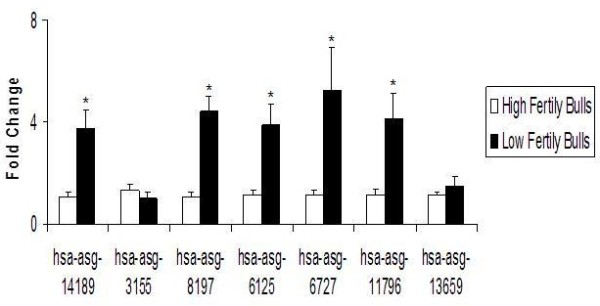
**Expression dynamics of seven miRNAs in sperm from six bulls with varying fertility.** Mean Ct values from the High fertility animals were used as reference points and Ct values for groups (high and low fertility) were used to calculate the fold change from the reference points for high fertility group using reference point Ct values according to 2-ΔΔCt method as described by Livak and Shimitgen [25]. Data were presented as mean value ± SEM for each miRNA and * indicates statistical significance at p < 0.05.

Real time RT-PCR analyses confirmed that miRNA expressions of hsa-asg-14189 (3.6 times), hsa-asg-8197 (4.5 times), hsa-asg-6125 (3.6 times), hsa-asg-6727 (3.9 times), and, hsa-asg-11796 (3.5 times) shows they were more abundant in the low fertility group when compared to the high fertility group (p < 0.0001, Figure 4). In contrast, the transcript levels of hsa-asg-3155 and hsa-asg-13659 did not show any difference between low fertility and high fertility groups by Real time RT-PCR.

## Discussion

MicroRNAs play an essential part of animal gene regulatory networks and a given genome could encode nearly 1000 miRNAs [20,26]. These short transcripts provide functions, in animals, that are essential for normal development and cellular homeostasis [27]. In this study, we identified miRNAs present in bovine spermatozoa. While examining two groups of bulls having different fertility we found that there was an abundance of miRNAs in bovine spermatozoa. However, only a few (seven) miRNAs were significantly differentially expressed. These seven miRNAs had a probe set which were from *Homo sapiens*. This could possibly mean that the sequences are conserved through species of *Bos taurus* and *Homo sapiens* although some miRNAs appear to be species specific [20]. However, among the seven significantly expressed miRNAs that were identified, there were none with published annotations; therefore, at the present time, their functions are unknown. Nevertheless, some studies have given explanations for possible functions of miRNAs through overexpression, misexpression, and *in vitro* knockdown [28].

In our studies, we observed that miRNAs detected in low fertility bull were expressed at higher levels than those in the high fertility bull. The higher expression levels of the miRNA in the low fertility bulls could mean that miRNAs might be down regulating expression of genes whose products play important roles in fertilization and early embryonic development. For example, recently it has been demonstrated that sperm borne miRNAs have been transferred into the oocyte and that miR34c is involved in the first cleavage division in mouse [29]. In this study we found that there were some variations of miRNA expressions within the same groups of bulls. This could possibly be due to the collection times, other variables such as nutrition or animal health, or technical variations. Semen was collected within the three month period and shipped to our laboratory. It is possible that gene-environment interactions among other factors might contribute to differences in gene expression including miRNAs in different ejaculates. Another limitation was that the miRNA microarray contained probes for known miRNAs for humans, mice and rats. Thus, it is expected that additional and bovine specific miRNAs would have been detected using bovine specific miRNA microarrays if they had been available. In spermatogenesis, there are many miRNAs that are waiting to be detected but little is known about their expression level or patterns [30].

Molecular interactions contribute to the homeostasis and the performance of a cell. Networking between genes, RNA, proteins and other biological molecules represent the interactome of a cell. Interactomic approach sheds light on miRNA as a regulator of networking proteins and will give the functional annotations of the molecules involved. Because of the interconnectivity among other proteins and the expression of miRNA (hsa-aga-6125) against BTBD2, it is possible that this miRNA might be the master regulator for expression of this set of genes. Co-expression of the miRNAs might be regulating the physical interactions among the networks of genes such as CHN2-EPHA1-EFNA2 (Tables 3 and 4). The target gene, AQP7P1 of probe ID hsa-aga-13659 is a member of the Aquaporin gene family. It encodes for Aquaporin 7 pseudogene 1, an integral membrane protein playing a role as a channel for aqueous flow across the membrane. Aquaporin 7 (AQP7) is known to be expressed in human sperm tails and lack of its expression has also been observed in some infertile patients. AQP7 positive sperm have significantly higher motility rate than that of AQP7 negative sperm, making this gene expression one of the deciding factors for male infertility [31]. IFT80 gene expression is determined to be targeted by hsa-miR-15b_st1. This gene product is a component of the intraflagellar transport complex which is essential for the development of motile cilia [32]. Probe ID involved in Dynamin 2 (DNM2) protein regulation is hsa-miR-638_st2. Dynamin 2 is found in the acrosomal region of the sperm, indicating its possible role during fertilization [33].

To what extend sperm miRNAs target sperm mRNAs? For this quest, we looked for targets of the top 10 miRNAs in the sperm mRNA data reported in the literature. In this present study we are reporting the genes that are the predicted targets of miRNAs which are listed in the Tables 3and 4. We searched target sperm mRNAs in the current literature for our target genes that were led by the top 10 miRNAs and we did not find any matches [2,34,35]. Therefore, the genes that we are reporting in this paper may have been potential targets for an extended study for a sperm mRNA focused study.

It is clear that miRNAs are abundant in bovine spermatozoa. Determining the function(s) of the significantly differentially expressed miRNAs will help us understand functional genomics of bovine spermatozoa. Distinct bovine miRNAs have been identified in bovine adipose tissue that should be useful for studying the role of miRNAs in cattle and for comparative genomic analysis of miRNA function and regulations [17]. Identifying specific miRNAs expressed in spermatozoa of different fertility, will help to better understand mammalian gametogenesis. From this we will be able to develop a model which will aid in understanding the roles of miRNAs during early development both in other animals, as well as, mammals including humans and bovine studied here. It would be interesting to identify target mRNAs of these miRNAs detected in bull spermatozoa. This study provides the groundwork for uncovering the role of miRNA in differential fertility. Future studies will include target prediction for the identified spermatozoal miRNA and thus develop a miRNA based molecular biomarker for fertility.

## Conclusions

In addition to providing half of the genomic DNA, research shows that the male gamete harbors large amounts of miRNAs. These small transcripts may have roles in sperm physiology including fertilization, egg activation and embryo development.

## Competing interests

Authors declare that they have no competing interests in this study.

## Authors' contributions

EM designed the study; AG did bioinformatics analyses and helped write the paper; LR helped write the paper; OA performed the real time RT-PCR, AK and ET provided sperm from high and low fertility bulls, provided the bull fertility data, and helped write the paper; EAC helped with data analyses, bioinformatics and manuscript writing; AU, JP and AP performed bioinformatics analyses and helped write the paper. All authors read and approved the final manuscript.

## Supplementary Material

Additional file 1 Table S1. Complete microarray output for the samples. Click here for file
